# GEOGRAPHIC DISPARITIES IN COUNTY-LEVEL PREVALENCE OF ALZHEIMER’S DISEASE ACROSS THE UNITED STATES

**DOI:** 10.1093/geroni/igz038.2878

**Published:** 2019-11-08

**Authors:** Mackenzie E Fowler, Christina DiBlasio, Michael Crowe, Richard E Kennedy

**Affiliations:** University of Alabama at Birmingham, Birmingham, Alabama, United States

## Abstract

Small-area geographic disparities in health care delivery have been observed across multiple disorders, but remain poorly studied for Alzheimer’s disease (AD) and other dementias. While national and state estimates of the prevalence and incidence of AD are available, estimates across finer geographic regions offer an opportunity to tailor programs to the needs of the local population. We estimated prevalence of AD at the county level across the continental United States. We used prevalence rates of AD by age category and race among Medicare fee-for-service beneficiaries published by the Centers for Disease Control (CDC). These prevalence rates were projected onto bridged-race county-level population data for 2017 from the National Center for Health Statistics, with empirical Bayes spatial smoothing to reduce variability in rates due to small population sizes. Estimated prevalence of AD varied more than threefold across counties, from a low of 51.8 per 1,000 persons in Loving County, Texas to a high of 169.6 per 1,000 persons in Kalawao County, Hawaii. Higher prevalence of AD was seen in the Southeastern and Midwestern United States. However, we observed specific counties with low prevalence of AD within regions with high prevalence of AD, and vice versa. These small-area geographic variations may provide vital information about social and environmental influences on dementia care, yet little data have been available to date. Understanding geographic disparities in prevalence will be critical for addressing practice variation in the prevention and diagnosis of dementia.

